# The Pan American Medical Congress

**Published:** 1893-03

**Authors:** 


### THE PAN-AMERICAN MEDICAL CONGRESS.

At the meeting of the American Medical Association in Detroit last May resolutions were passed extending a "cordial invitation to the medical profession of the Western hemisphere to assemble in the United States in an nter-continental American Medical Congress." A permanent organization of the proposed Congress was effected, and the first meeting of the same was appointed for Washington, September 5th to 8th, 1893. This Medical Congress will be one of the most important that will have assembled on this continent, second only to the International Congress of six years ago. Reports and abstracts of papers will be published in the English, French, Spanish and Portuguese languages. Those who expect to present papers at the meeting should forward an abstract of the same, not exceeding six hundred words, to the Secretary-General, by the 10th of July, 1893. These abstracts will be published in the above languages prior to the meeting. The membership fee will be $10 for doctors residing in the United States; no fee will be required from foreign members. The officers of the Congress are: President, Dr. William Pepper, Philadelphia; Secretary-General, Dr. Chas. A. L. Reed, Cincinnati, Ohio; Treasurer, Dr. A. M. Owen, Evansville, Ind.; Chairman of Committee of Arrangements, Dr. Samuel S. Adams, Washington, D. C. All the officers are deeply interested in this Congress, and are pushing it with vigor and determination. We hope it will be well attended and productive of great and useful results.

